# Dietary Omega-3 Fatty Acids Promote Arrhythmogenic Remodeling of Cellular Ca^2+^ Handling in a Postinfarction Model of Sudden Cardiac Death

**DOI:** 10.1371/journal.pone.0078414

**Published:** 2013-10-18

**Authors:** Andriy E. Belevych, Hsiang-Ting Ho, Radmila Terentyeva, Ingrid M. Bonilla, Dmitry Terentyev, Cynthia A. Carnes, Sandor Gyorke, George E. Billman

**Affiliations:** 1 Dorothy M. Davis Heart and Lung Research Institute, the Ohio State University, Columbus, Ohio, United States of America; 2 Department of Physiology and Cell Biology, College of Medicine, the Ohio State University, Columbus, Ohio, United States of America; 3 College of Pharmacy, the Ohio State University, Columbus, Ohio, United States of America; 4 Department of Medicine, Rhode Island Hospital and the Warren Alpert Medical School of Brown University, Providence, Rhode Island, United States of America; Rutgers-New Jersey Medical School, United States of America

## Abstract

It has been proposed that dietary omega-3 polyunsaturated fatty acids (n-3 PUFAs) can reduce the risk of ventricular arrhythmias in post-MI patients. Abnormal Ca^2+^ handling has been implicated in the genesis of post-MI ventricular arrhythmias. Therefore, we tested the hypothesis that dietary n-3 PUFAs alter the vulnerability of ventricular myocytes to cellular arrhythmia by stabilizing intracellular Ca^2+^ cycling. To test this hypothesis, we used a canine model of post-MI ventricular fibrillation (VF) and assigned the animals to either placebo (1 g/day corn oil) or n-3 PUFAs (1-4 g/day) groups. Using Ca^2+^ imaging techniques, we examined the intracellular Ca^2+^ handling in myocytes isolated from post-MI hearts resistant (VF-) and susceptible (VF+) to VF. Frequency of occurrence of diastolic Ca^2+^ waves (DCWs) in VF+ myocytes from placebo group was significantly higher than in placebo-treated VF- myocytes. n-3 PUFA treatment did not decrease frequency of DCWs in VF+ myocytes. In contrast, VF- myocytes from the n-3 PUFA group had a significantly higher frequency of DCWs than myocytes from the placebo group. In addition, n-3 PUFA treatment increased beat-to-beat alterations in the amplitude of Ca^2+^ transients (Ca^2+^ alternans) in VF- myocytes. These n-3 PUFAs effects in VF- myocytes were associated with an increased Ca^2+^ spark frequency and reduced sarcoplasmic reticulum Ca^2+^ content, indicative of increased activity of ryanodine receptors. Thus, dietary n-3 PUFAs do not alleviate intracellular Ca^2+^ cycling remodeling in myocytes isolated from post-MI VF+ hearts. Furthermore, dietary n-3 PUFAs increase vulnerability of ventricular myocytes to cellular arrhythmia in post-MI VF- hearts by destabilizing intracellular Ca^2+^ handling.

## Introduction

Cardiac arrhythmias are recognized as a major factor contributing to morbidity and mortality in patients with healed myocardial infarction (MI). The search for an effective anti-arrhythmic therapy remains a major unmet challenge. Initial observational and interventional studies indicated that dietary omega-3 polyunsaturated fatty acids (n-3 PUFAs) may be effective in preventing cardiac arrhythmias [1–3]. However, more recent clinical and animal studies reported mixed results as to the anti-arrhythmic effects of n-3 PUFAs [4–7]. To explain the apparent heterogeneity of the results, it has been suggested that the effectiveness of n-3 PUFAs treatment might depend on the mechanism of cardiac arrhythmia (triggered vs. reentry), and on the route of n-3 PUFAs administration (infused, free circulating vs. dietary, lipid incorporated)[6,8].

Abnormal regulation of intra-myocyte Ca^2+^ handling observed in various cardiac disease settings, including post-MI hearts, has been implicated in the genesis of both triggered and reentrant arrhythmias [9–13]. Mechanistically, dysregulation of Ca^2+^cycling that is manifested by increased frequency of diastolic Ca^2+^ waves (DCWs) and Na^+^/Ca^2+^ exchanger-mediated delayed after-depolarizations (DADs) is usually associated with triggered arrhythmia mechanisms. Additionally, remodeling of Ca^2+^ handling that results in increased susceptibility to beat-to-beat alterations in the amplitude of Ca^2+^ transients (Ca^2+^ alternans), and thereby an increased dispersion of repolarization, can be linked to reentrant mechanisms of arrhythmia. Therefore, the overall success of anti-arrhythmic treatment with n-3 PUFAs may depend upon its effects on intra-myocyte Ca^2+^ handling.

In cellular studies, the acute application of free n-3 PUFAs consistently depressed intracellular Ca^2+^ handling, by reducing the frequency Ca^2+^ sparks [14], Ca^2+^ after-transients [15] and Ca^2+^ influx via the L-type Ca^2+^ channels [16–19], as well as decreasing levels of systolic and diastolic Ca^2+^ [15,17,19], and inhibiting the activity of reconstituted ryanodine receptors (RyR2s) [14,20], a sarcoplasmic reticulum Ca^2+^ release channel. These data indicate that free n-3 PUFAs can be effective in suppressing diastolic Ca^2+^ waves and in preventing triggered arrhythmia [15,19]. Similarly, dietary n-3 PUFAs were shown to suppress arrhythmic contractile activity and Ca^2+^ after-transients in myocytes isolated from control hearts [21,22]. However the effects of chronic dietary n-3 PUFAs on intracellular Ca^2+^ handling in diseased myocytes remain to be determined.

In the present study, we used a well-characterized canine model of healed MI [23] to investigate the effects of dietary n-3 PUFAs (1-4 g/day docosahexaenoic acid + eicosapentaenoic acid ethyl esters) on intracellular Ca^2+^ cycling in isolated ventricular myocytes. Using a standardized exercise plus ischemia test, post-MI animals were stratified for susceptibility to ventricular fibrillation (VF) into susceptible (VF+) and resistant (VF-) groups. We show that dietary n-3 PUFAs produced alterations in intracellular Ca^2+^ cycling in post-MI myocytes that are consistent with a pro- rather than an anti-arrhythmic effect. 

## Materials and Methods

The principles governing the care and use of animals as expressed by the Declaration of Helsinki, and as adopted by the American Physiological Society, were followed at all times during this study. In addition, the Ohio State University Institutional Animal Care and Use Committee approved all the procedures used in this study.

### Model

A description of the model, n-3 PUFA treatment protocol, and previous *in vivo* results have been described in detail [7]. Briefly, heartworm free mixed breed dogs (2-3 y old) were anesthetized and instrumented to measure a ventricular electrogram and coronary blood flow as previously described [23–25]. A hydraulic vascular occluder was placed around the left circumflex coronary artery and used to induce acute myocardial ischemia during the exercise plus ischemia test as described below. The left anterior descending coronary artery was also isolated during the instrumentation surgery and a two-stage occlusion of this artery was then performed approximately one-third the distance from its origin in order to produce an anterior wall myocardial infarction (~16% of left ventricular mass [23]). Three-to-four weeks after the production of the myocardial infarction, the susceptibility to ventricular fibrillation (VF) was tested as previously described [23–25]. The animals ran on a motor-driven treadmill while workload progressively increased until a heart rate of 70% of maximum (approximately 210 beats/min) had been achieved. During the last minute (on average during the 18^th^ minute) of exercise, the left circumflex coronary artery was occluded, the treadmill stopped and the occlusion maintained for an additional minute (total occlusion time = 2 min.). The exercise plus ischemia test reliably induced ventricular flutter that rapidly deteriorated into VF. Therefore, large defibrillation electrodes were placed across the animal’s chest so that electrical defibrillation could be achieved with a minimal delay but only after the animal was unconscious (10-20 s after the onset of VF). The occlusion was immediately released if VF occurred. 

### Omega-3 protocol

The dogs were placed on a diet that did not contain any n-3 PUFAs beginning one week prior to the instrumentation surgery and were maintained on this diet until the end of the study (~ 4 months). After the pre-treatment data collection (3 - 4 weeks after the surgery), the dogs were then randomly assigned to the following groups: placebo (n = 17: VF+, n = 9; VF-, n = 8); n-3 PUFA (1-4 g/day, n = 45: VF+ n = 22; VF-, n = 23). The dogs were given supplements similar to those used in the GISSI-Prevenzione study [26]. The n-3 PUFA group received 465 mg ethyl eicosapentaenoate, EPA + ethyl docosahexaenoate, DHA, 375 mg per 1 g capsule (Lovaza®, GlaxoSmithKline, Research Triangle Park, NC); doses of 1, 2, 4 grams were given. As no dose-dependent differences were found, data for all doses were grouped together. The placebo was corn oil (1 g, 58% linoleic acid + 28% oleic acid). The capsules were given *per os* prior to the daily feeding (between 8:00 and 10:00 AM each day, 7 days per week for 3 months). As previously reported [7,27], dietary EPA +DHA ethyl esters elicited significant increases in left ventricle n-3 PUFA content, reaching a peak between 8 and 12 weeks. 

### Cellular Ca^2+^ imaging

Myocytes were isolated distant from the infarction zone of the left ventricular midmyocardium as described previously [28]. For present study cells were isolated from normal control dogs (n=8, no surgery, no MI, untreated), n-3 PUFAs treated sham controls (n=3, no MI), untreated VF- (n=2), placebo treated VF- (n=3) and VF+ (n=3) dogs, and n-3 PUFAs treated VF- (n=3) and VF+ (n=4) dogs. Electrical field stimulation experiments were performed using the following external solution (in mM): 140 NaCl, 5.4 KCl, 2.0 CaCl_2_, 0.5 MgCl_2_, 10 HEPES, and 5.6 glucose (pH 7.4). Intracellular Ca^2+^ imaging was performed using an Olympus Fluoview 1000 confocal microscope. Rhod-2 Ca^2+^ indicator was used to monitor cytosolic Ca^2+^ in intact myocytes. Cells were incubated with 10 µM Rhod-2 AM (Life Technologies, Grand Island, NY) for 25 min at room temperature. Amplitude of Ca^2+^ alternans was defined as 100-(A_2_/A_1_)*100 (%), where A_1_ and A_2_ are amplitudes of two consecutive Ca^2+^ transients. Ca^2+^ sparks were studied in saponin-permeabilized myocytes using 30 µM Fluo-3 (Life Technologies, Grand Island, NY) and the following intracellular solution: (mM) 120 potassium aspartate, 20 KCl, 3 MgATP, 10 phosphocreatine, 5 U ml^-1^ creatine phosphokinase, 0.5 EGTA (pCa 7) and 20 HEPES (pH 7.2). Ca^2+^ sparks were detected and analyzed using a computer algorithm described previously [29]. Image processing and analysis was performed using ImageJ (National Institutes of Health; http://rsbweb.nih.gov/ij/) and Origin 7.0 (OriginLab Corporation, Northampton, MA) programs. 

### Western Blotting

The levels of proteins involved in Ca^2+^ cycling and their phosphorylation were assessed by immunoblot analysis using 20-40 mg of homogenates from left ventricular tissue samples as described previously [30]. Primary antibodies used were: anti-phospholamban(PLB), anti- Na^+^/Ca^2+^ exchanger(NCX1), and anti-phospho-PLB-S16 from Millipore (Billerica, MA); anti-SERCA2a from Sigma-Aldrich (St.Luis, MO); anti-RyR2 and anti-Cav1.2 from ThermoScientific (Waltham, MA); anti-phospho-PLB-T17 from Santa Cruz (Dallas, TX). Anti-phospho-RyR2-S2030 antibody was raised against (CG) TIRGRLLS(PO4)LVEKVTYLKKCONH2 (YenZym Abs, South San Francisco, CA). Custom-made anti-phospho-RyR2-S2808 and anti-phospho-RyR2-S2814 were from Phosphosolutions (Aurora, CO)[30]. Anti- glyceraldehyde 3-phosphate dehydrogenase (GAPDH) antibody was from Abcam (Cambridge, MA). Expression levels of RyR2, SERCA2A, PLB, Cav1.2 channels, and NCX1 were assessed after normalization to the loading control, GAPDH. Phosphorylation levels of RyR2 and PLB were analyzed following normalization to RyR2 or PLB protein levels assessed from gels run in parallel. Blots were developed with Super Signal West Pico (Pierce) and quantified using ImageJ (National Institutes of Health) and Origin 7 (OriginLab, Northampton, MA) software. 

### Statistical Analysis

Results are presented as mean±S.E.M. Statistical significance was evaluated using either Student's t test or one way ANOVA with Tukey’s post hoc test. The proportion of cells displaying DCWs or Ca^2+^ alternans was compared using Fisher's exact test. A P value of <0.05 was considered significant.

## Results

### Dietary n-3 PUFAs do not stabilize intracellular Ca^2+^ cycling in VF+ myocytes and increase susceptibility of VF- myocytes to pro-arrhythmic diastolic Ca^2+^ waves

**Figure 1:**
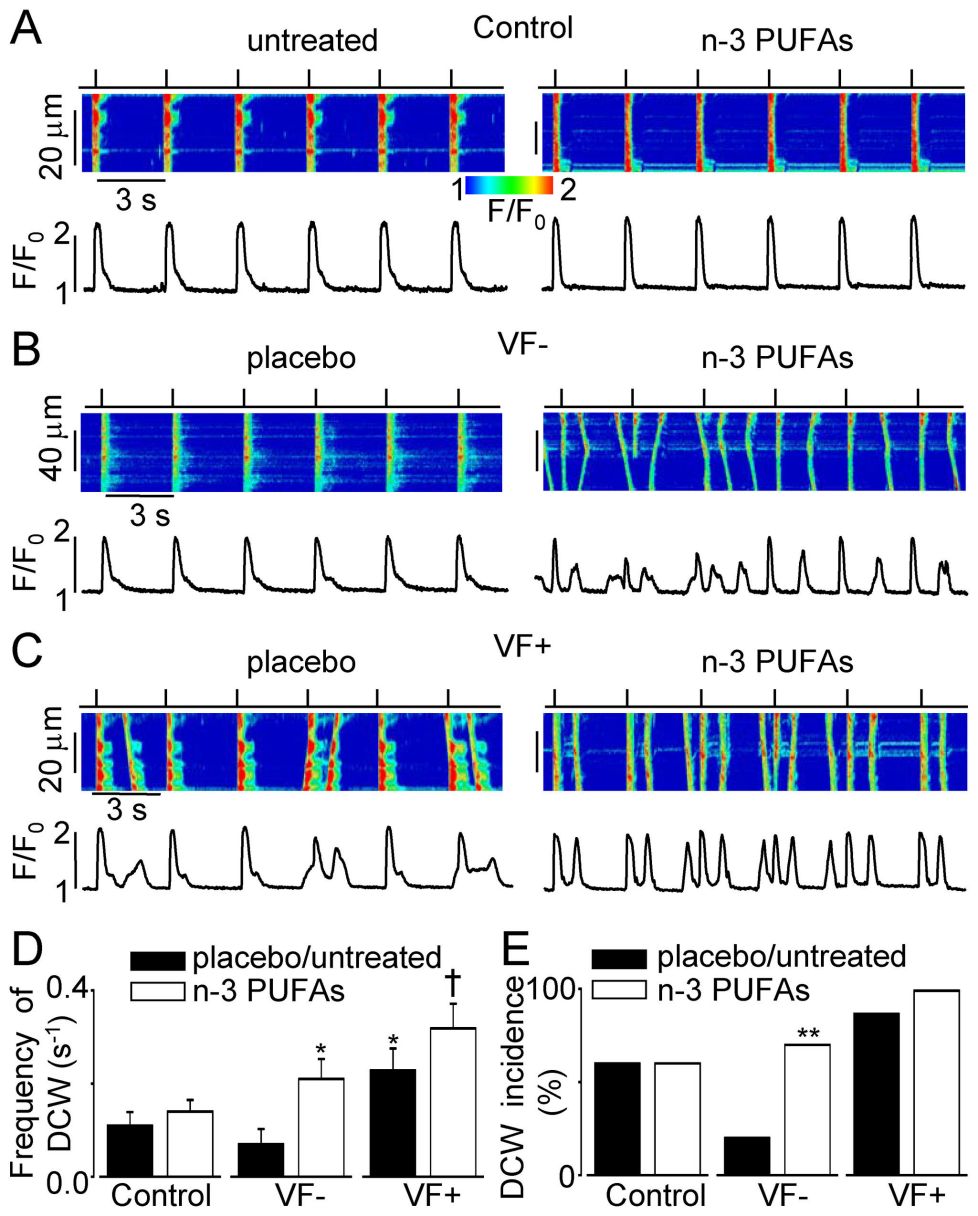
Dietary n-3 PUFAs induce pro-arrhythmic remodeling of intracellular Ca^2+^ handling in VF- myocytes. Representative line-scan images and corresponding profiles of Rhod-2 fluorescence during periodic (0.3 Hz) electrical stimulation recorded in myocytes from placebo/untreated and n-3 PUFA-treated controls (**A**), VF- (B) and VF+ (C) groups, respectively. Data were obtained in the presence of 100 nM isoproterenol, a β-adrenergic receptor agonist. **D**, Average frequency of DCWs (per second) was: 0.11±0.03 (n=15) and 0.14±0.03 (n=40) in control untreated and n-3 PUFA-treated myocytes, respectively (P=0.5); 0.07±0.03 (n=20) and 0.21±0.04 (n=20) in VF- myocytes from placebo and n-3 PUFAs groups, respectively (P=0.014); 0.23±0.05 (n=8) and 0.32±0.05 (n=8), in VF+ myocytes from placebo and n-3 PUFAs groups, respectively (P=0.22). *, P<0.05 *vs*. VF- placebo; †, P<0.05 *vs*. n-3 PUFA-treated controls. **E**, Bar graph shows proportion of myocytes displaying DCWs. In control groups DCWs were recorded in 9 out of 15 untreated cells and in 24 out of 40 n-3 PUFA-treated cells, respectively. In VF- groups DCWs were recorded in 4 out of 20 placebo-treated cells and in 14 out of 20 n-3 PUFA-treated cells, respectively. In VF+ groups DCWs were recorded in 7 out of 8 placebo-treated cells and in 8 out of 8 n-3 PUFA-treated cells, respectively.

Recordings of cytosolic Ca^2+^ in field-stimulated myocytes in the presence of β-adrenergic receptor agonist isoproterenol (100 nM) were used to analyze susceptibility of ventricular myocytes to DCWs. On average, the frequency of occurrence of DCWs was not different in untreated controls and VF- myocytes from placebo group (Figure 1 *A, B*, *D*). DCWs were more frequent (P<0.05) in field-stimulated VF+ myocytes from the placebo-treated group than in VF- myocytes from corresponding group (Figure 1 B-E). n-3 PUFA treatment did not affect the rate of the occurrence of DCWs either in control (P=0.5) or in VF+ (P=0.2) myocytes (Figure 1 *A, C*, *D, E*). Conversely, in VF- myocytes n-3 PUFA treatment significantly increased (P<0.05 *vs.* placebo) frequency of DCWs (Figure 1 B, D). Furthermore, the proportion of myocytes displaying DCWs increased more than three-fold (P<0.01) in VF-myocytes treated with n-3 PUFAs when compared to placebo-treated cells (Figure 1 E). 

### Dietary n-3 PUFAs increase susceptibility of VF- myocytes to pro-arrhythmic Ca^2+^ alternans

**Figure 2:**
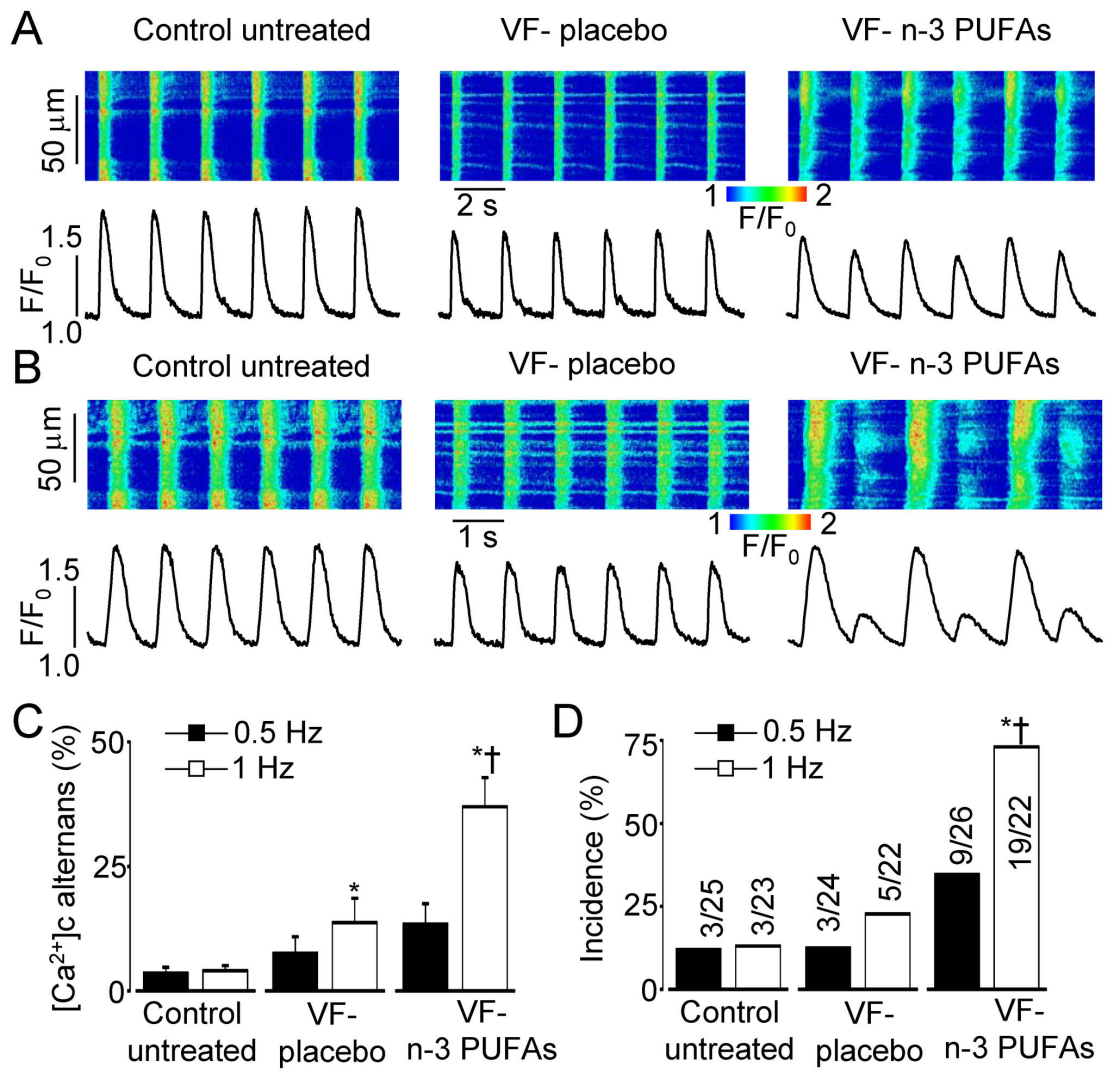
Dietary n-3 PUFAs increase susceptibility of VF- myocytes to pro-arrhythmic Ca^2+^ alternans. Representative line-scan images and corresponding profiles of Rhod-2 fluorescence recorded in myocytes from indicated groups at 0.5 (**A**) and 1 Hz (**B**) stimulation. Bar graphs show amplitude (**C**) and incidence (**D**) of Ca^2+^ alternans recorded in control untreated myocytes and VF- myocytes from placebo and n-3 PUFAs groups at 0.5 and 1Hz. Number of myocytes with amplitude of Ca^2+^ alternans larger than 10 % (numerator) and total number of myocytes studied (denominator) are indicated for each group presented in panel D bar graph. *, P<0.05 *vs*. control (1Hz); †, P<0.05 *vs*. VF- placebo (1Hz).

To investigate whether the effects of dietary n-3 PUFAs on VF- myocytes were associated with Ca^2+^-dependent arrhythmogenic substrate, we studied the amplitude and rate-dependence of Ca^2+^ alternans in VF- myocytes from placebo and n-3 PUFA group [9,31]. As demonstrated in Figure 2, both untreated controls and placebo-treated VF- myocytes did not normally exhibit Ca^2+^ alternans at 0.5 and 1 Hz frequency of field stimulation. In contrast, following n-3 PUFA treatment 75 % of VF- myocytes displayed Ca^2+^ alternans at 1 Hz (Figure 2 B). This increase in a number of cells displaying alternans was also associated with a significant increase (P<0.05 *vs.* placebo) in average amplitude of Ca^2+^ alternans recorded in VF- from n-3 PUFAs treated group at 1 Hz (Figure 2 *B, C*, *D*). These data suggest that dietary n-3 PUFAs may enhance the dynamic substrate for arrhythmia in VF- hearts. 

### Effect of dietary n-3 PUFAs on intracellular Ca^2+^ handling in VF- myocytes is associated with the increased ryanodine receptor (RyR2) activity

**Figure 3:**
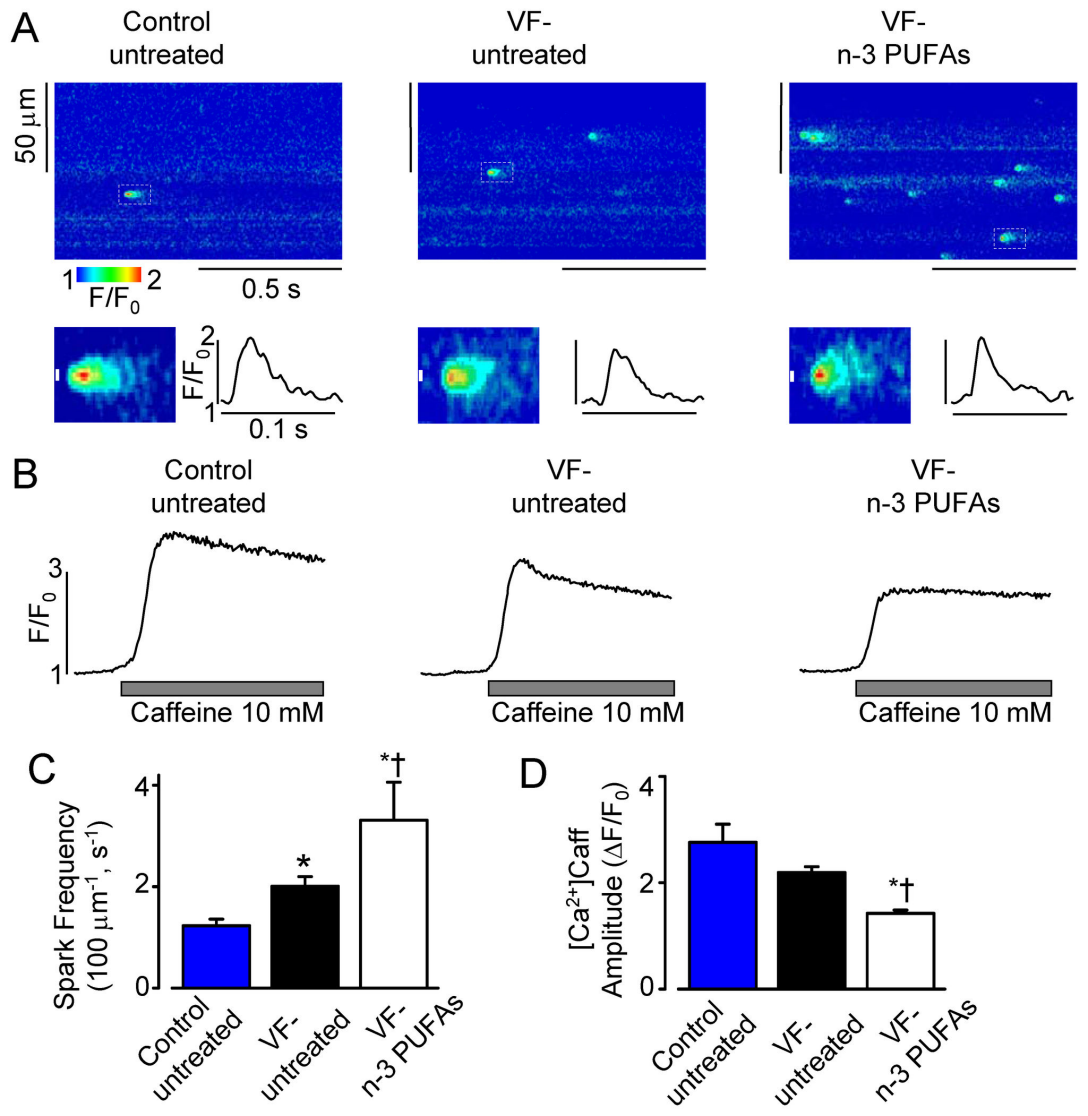
Dietary n-3 PUFAs increase frequency of Ca^2+^ sparks in VF- myocytes. **A**, Representative line-scan images of Ca^2+^ sparks recorded in saponin-permeabilized myocytes from indicated groups. Insets show scaled up image of Ca^2+^ spark with corresponding time-dependent fluorescence profile. **B**, Representative traces of Ca^2+^ transients evoked by 10 mM caffeine recorded in permeabilized myocytes from indicated groups. **C**, Average Ca^2+^ spark frequency (in 100 µm^-1^*s^-1^) was 1.23±0.13 (n=52) in untreated control myocytes, 2.01±0.19 (n=47) and 3.31±0.38 (n=45) in untreated and n-3 PUFA-treated VF- myocytes, respectively. **D**, Average amplitude of caffeine-induced Ca^2+^ transients ([Ca^2+^]_CAFF_, ΔF/F_0_) was 2.76±0.34 (n=7) in untreated control myocytes, 2.19±0.11 (n=5) and 1.42±0.06 (n=4) in untreated and n-3 PUFA-treated VF- myocytes, respectively. *, P<0.05 *vs*. control; †, P<0.05 *vs*. VF- untreated.

We further characterized the effect of dietary n-3 PUFAs on properties of intracellular Ca^2+^ handling in VF- myocytes by measuring the frequency of Ca^2+^ sparks. As shown in Figure 3 and Table 1 Ca^2+^ sparks frequency was significantly higher in untreated VF- myocytes when compared to control. However, even greater increases in Ca^2+^ spark frequency were observed in VF- myocytes from the n-3 PUFA treated group (Figure 3 A, C; table 1). To assess possible mechanisms underlying the n-3 PUFA-induced augmented Ca^2+^ spark activity in VF- myocytes, we studied SR Ca^2+^ content ([Ca^2+^]_SR_) by measuring the amplitude of Ca^2+^ transients evoked by 10 mM caffeine. As shown in Figure 3 (B and D), [Ca^2+^]_SR_ was significantly lower in n-3 PUFA-treated VF- myocytes compared to untreated control and VF- myocytes, respectively. More frequent Ca^2+^ sparks at lower [Ca^2+^]_SR_ indicate increased RyR2 functional activity in VF- myocytes from n-3 PUFA-treated group.

**Table 1:** Properties of Ca^2+^ sparks in saponin-permeabilized ventricular myocytes.

	Control untreated *n=52*	VF- untreated *n=47*	VF- n-3 PUFAs *n*=45
Amplitude (ΔF/F_0_)	0.74±0.02	0.64±0.01*	0.70±0.01^†^
Frequency (sparks/100 µm/s)	1.23±0.13	2.01±0.19*	3.31±0.38*^†^
FDHM (ms)	21.9±0.8	23.8±0.8	18.0±0.4*^†^
FWHM (µm)	2.07±0.05	1.96±0.04	2.06±0.04
Time to peak (ms)	10.50±0.39	9.71±0.43	8.32±0.27*^†^

FDHM, full duration at half maximum; FWHM, full width at half maximum. N, number of cells studied. * P<0.05 *vs.* control untreated; ^†^ P<0.05 *vs.* VF- untreated.

**Figure 4:**
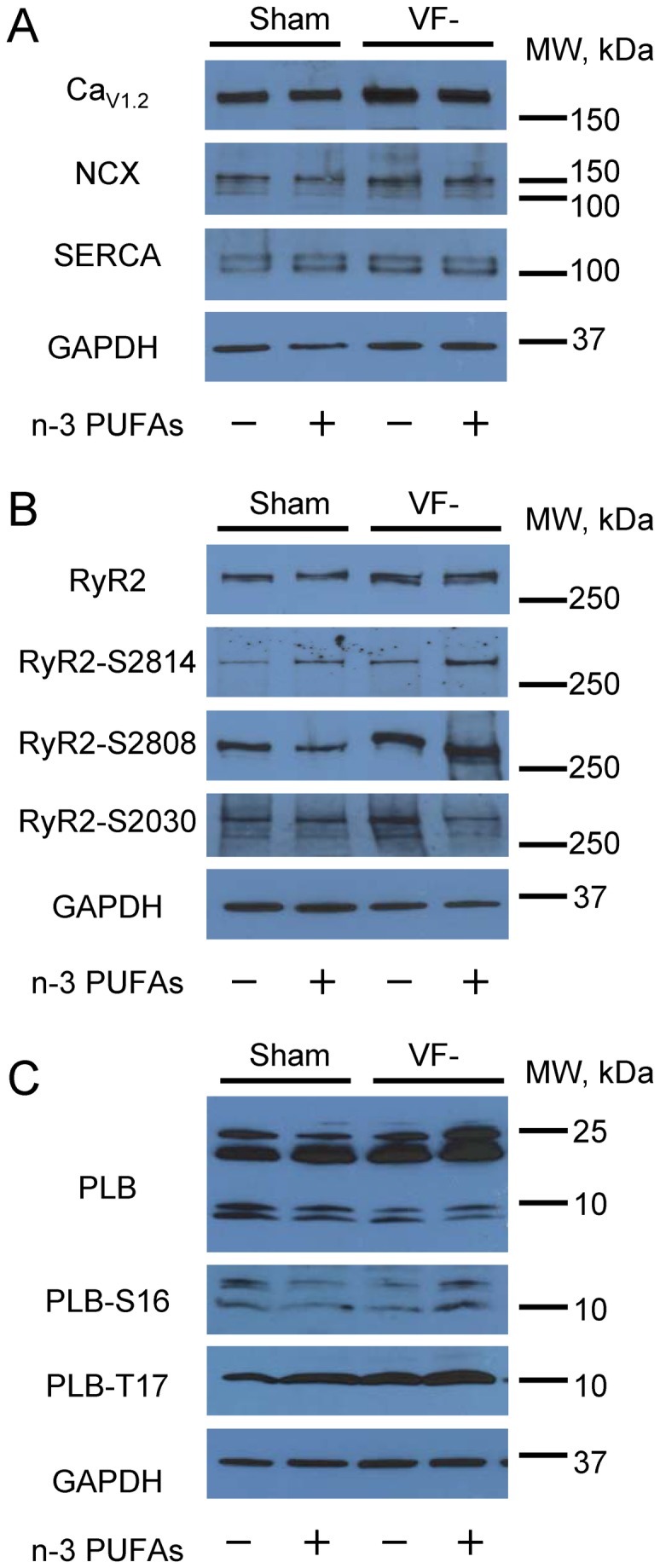
Dietary n-3 PUFAs do not affect expression and phosphorylation levels of proteins involved in cardiac Ca^2+^ cycling. **A**-**C**, Representative immunoblots of left ventricle homogenates prepared from placebo and n-3 PUFA-treated control (sham) and VF- groups. NCX1, Na^+^/Ca^2+^ exchanger type 1; SERCA2a, cardiac isoform of SR Ca^2+^-ATPase; GAPDH, Glyceraldehyde 3-phosphate dehydrogenase; PLN, phospholamban; Cav1.2, α1C subunit of L-type Ca^2+^ channel. Data were obtained using 2-4 heart samples. Quantitative analysis is presented in Table 2.

Next, we assessed whether changes in expression and phosphorylation levels of proteins involved in intracellular Ca^2+^ cycling occur following chronic dietary supplementation with n-3 PUFAs. Dietary n-3 PUFAs did not significantly affect expression of RyR2, SR Ca^2+^ ATPase, phospholamban (PLB), alpha subunit of cardiac L-type Ca^2+^ channels, and Na^+^/Ca^2+^ exchanger either in control or in VF- ventricular preparations (Figure 4 A-C, table 2). We also observed no significant alterations in RyR2 phosphorylation at well-established phosphorylation sites (Ser-2808, Ser-2814, and Ser-2030)[32,33] in n-3 PUFA-treated groups (Figure 4 B, table 2). Finally, phosphorylation levels of PLB at Ser-16 and Thr-17 were also unaffected by n-3 PUFA treatment (Figure 4 C, table 2).

**Table 2:** Expression and phosphorylation levels of critical Ca^2+^ handling proteins in n-3 PUFA-treated control and VF- preparations.

	Control n-3 PUFAs (Normalized to control placebo, %)	VF- n-3 PUFAs (Normalized to VF- placebo, %)
RyR2	132±18	116±34
RyR2-S2808	75±7	115±27
RyR2-S2814	180±47	97±29
RyR2-S2030	111±26	95±22
SERCA2A	98±35	149±29
PLB	90±7	88±11
PLB-S16	125±49	365±72
PLB-T17	158±19	125±12
Cav1.2	135±33	145±49
NCX	87±20	139±53

Data were obtained from 2-4 experiments. No significant differences (P>0.05) were observed in protein expression or protein phosphorylation levels between placebo and n-3 PUFAs treatments.

## Discussion

In the present study we tested the hypothesis that dietary n-3 PUFAs would stabilize intracellular Ca^2+^ cycling in ventricular myocytes isolated from post-MI hearts. The major findings are as follows: a) dietary n-3 PUFAs were not effective in inhibiting DCWs in ventricular myocytes isolated from VF+ animals; b) dietary n-3 PUFAs caused marked destabilization of intracellular Ca^2+^ cycling in myocytes from VF- animals manifested as an increased rate of occurrence of DCWs and increased amplitude of Ca^2+^ alternans; c) effects of dietary n-3 PUFAs observed in VF- myocytes were associated with enhanced RyR2 activity. These cellular findings may explain our previous *in vivo* observation that dietary n-3 PUFA not only failed to reduce the risk for ventricular tachyarrhythmias in VF+ dogs but actually increased arrhythmia formation in VF- dogs [7].

Intracellular Ca^2+^ dysregulation is recognized as an important factor contributing to the genesis of various forms of cardiac arrhythmias. Remodeling of intracellular Ca^2+^ cycling leading to increased occurrences of spontaneous Ca^2+^ releases and diastolic Ca^2+^ waves is typically associated with triggered arrhythmias [10,11,13]. Alterations in intracellular Ca^2+^ handling resulting in beat-to-beat variations in the amplitude of Ca^2+^ transient (Ca^2+^ alternans) are believed to contribute to reentrant excitation, providing an additional form of proarrhythmic dysregulation [9–11]. Using canine post-MI model of sudden cardiac death we previously showed that ventricular myocytes isolated from VF+ hearts had higher susceptibility to both DCWs [34] and Ca^2+^ alternans [31] when compared to myocytes isolated from normal hearts. In the present study using the same animal model, we investigated the effects of dietary n-3 PUFAs on intracellular Ca^2+^ cycling [23]. Dietary EPA +DHA ethyl esters supplements significantly increased left ventricular n-3 PUFA content [7,27]. The increased n-3 PUFA tissue content did not alter the already high propensity of VF+ myocytes for DCWs (Figure 1 C, D). Furthermore dietary n-3 PUFAs increased susceptibility of VF- myocytes to both DCWs and Ca^2+^ alternans (Figure 1 *A, B*, *D* and Figure 2 B-D). Although molecular mechanisms responsible for these effects of n-3 PUFAs remain to be determined, our cellular data demonstrate that incorporated n-3 PUFAs can be linked to increased susceptibility to both triggered and reentrant arrhythmias in post-MI hearts. 

It has been previously noted that the physiological effects of n-3 PUFAs might depend on the route of administration: acute application of free n-3 PUFAs vs. chronic dietary consumption that results in increases in both free circulating and lipid incorporated PUFAs [6,8]. Indeed, most cellular data supporting an anti-arrhythmic effect of PUFAs were obtained from studies that evaluated the effects of the acute application of free n-3 PUFAs. Thus, acute application of free n-3 PUFAs invariably resulted in inhibitory effects on membrane excitability and Ca^2+^ handling [14–19] [reviewed in 6,35]. Consistent with these *in vitro* studies, acute infusion of free n-3 PUFAs reduced *in vivo* susceptibility to VF in our canine post-MI model [36]. 

Animal studies addressing the effects of dietary n-3 PUFAs have produced more heterogeneous results [reviewed in 6,35]. For example, dietary n-3 PUFAs inhibited ischemia and reperfusion arrhythmias in rat hearts [37] but promoted arrhythmias during acute myocardial ischemia in pig hearts [38] and increased *in vivo* susceptibility to VF in dogs with healed MI [7]; the very same animals from which myocytes were obtained for the present studies. In ventricular myocytes isolated from control animals, incorporated n-3 PUFAs did not significantly affect Ca^2+^ transients under baseline conditions, but reduced both arrhythmogenic Ca^2+^ after-transients and arrhythmic contractile activity evoked by beta-adrenergic receptor stimulation [21,22]. We previously showed that incorporated n-3 PUFAs did not change Ca^2+^ transients under baseline conditions in myocytes isolated from post-MI canine hearts [27]. To the best of our knowledge, the present study is the first to address the effect of dietary n-3 PUFAs on arrhythmogenic properties of intracellular Ca^2+^ cycling in the setting of healed MI with known *in vivo* susceptibility to cardiac arrhythmias. In our experiments, dietary n-3 PUFAs resulted in severe pro-arrhythmic alterations in intracellular Ca^2+^ cycling in VF- myocytes (Figures 1-3), whereas susceptibility of VF+ myocytes to DCWs, already high in the placebo group, was not significantly affected by n-3 PUFAs (Figure 1 C-E). It is worthwhile to note that dietary n-3 PUFAs did not affect the stability of intracellular Ca^2+^ cycling in ventricular myocytes isolated from controls (Figure 1 *A, D*, *E*) suggesting that the pro-arrhythmic effect may depend on cellular substrate (magnitude and mechanisms of cellular remodeling due to MI).

The n-3 PUFA influence on ion channel activity has been attributed to the direct interactions with the channel proteins and indirect effects on membrane fluidity and intracellular signaling [5,6,8]. Given that the acute application of n-3 PUFAs inhibits RyR2s [14,20], enhanced activity of RyR2s observed in the present study most likely results from indirect effects. We did not find evidence that dietary n-3 PUFAs alter expression levels of proteins involved in cardiac Ca^2+^ cycling including RyR2 (Figure 4, table 2). Since phosphorylation of RyR2 has been implicated in abnormal increase of RyR2 activity in disease states [32,33] and acute application of n-3 PUFAs has been associated with activation of protein kinase A [39], we also studied the effects of dietary n-3 PUFAs on phosphorylation state of RyR2. As illustrated in Figure 4 (B) and table 2, phosphorylation of established RyR2 phosphorylation sites was not significantly altered by dietary n-3 PUFAs suggesting that proarrhythmic effects of dietary n-3 PUFAs are not associated with the increased RyR2 phosphorylation. Further research will be needed to determine molecular mechanisms linking dietary n-3 PUFA and abnormal RyR2 activity. 

### Study limitation

We acknowledge that present study has some limitations that could affect the interpretation of the results. Due to technical reasons all cellular experiments were performed at room temperature (22-24°C) and ambient O_2_ tension (~20%) in contrast to physiological temperature (37°C) and O_2_ tension (~5%). Therefore, our experimental conditions could influence dynamics of intracellular Ca^2+^ cycling, fluidity of the membrane and potentially could alter the intracellular effects of incorporated n-3 PUFAs. Finally, efficacy of incorporated n-3 PUFAs may be different in canine and human hearts. 

## Conclusions

In the present study, we have demonstrated that increases in left ventricle n-3 PUFA content mediated by dietary intake of EPA +DHA ethyl esters similar to those noted in patients [26,40] were associated with a significant increases in frequency of Ca^2+^ sparks in myocytes from post-MI (VF-) hearts. The increased frequency of Ca^2+^ sparks along with the reduced SR Ca^2+^ content observed in VF- myocytes suggest that incorporated n-3 PUFAs increased sensitivity of ryanodine receptors to SR Ca^2+^ in diseased hearts. We further demonstrated that dietary n-3 PUFA supplements were associated with a high predisposition of both VF- and VF+ myocytes to DCWs in response to β-adrenergic receptor stimulation. Thus, we conclude that incorporated n-3 PUFAs produce disturbances in Ca^2+^ cycling that would increase rather than decrease the risk for ventricular tachyarrhythmias in post-MI hearts. 
